# Karyotype Description of Two Andean Species of the *guarani* Group of *Drosophila* (Díptera: Drosophilidae) and Cytological Notes

**DOI:** 10.1093/jisesa/ieab032

**Published:** 2021-05-15

**Authors:** Doris Vela, Erika Villavicencio

**Affiliations:** Pontificia Universidad Católica del Ecuador, Facultad de Ciencias Exactas y Naturales, Laboratorio de Genética Evolutiva, Avenida 12 de Octubre 1076 y Roca, Quito, Ecuador

**Keywords:** chromosome, *guarani*, Ecuador, Neotropical region

## Abstract

The *guarani* group of *Drosophila* genus (Diptera: Drosophilidae) is formed by 24 species however the relationship of these species is not clear. In the present study are described the karyotypes of *Drosophila sachapuyu* Peñafiel and Rafael, 2018 and *Drosophila zamorana* Peñafiel and Rafael, 2018, two Andean species members of the *guarani* group. Mitotic chromosomes from cerebral ganglia of third stand larval were obtained by thermal shock and cell suspension techniques. The karyotype of *D. sachapuyu*, presents 2n = 10 (4R, 1V; X = R, Y = R) while *D. zamorana* exhibits karyotype 2n = 12 (5R, 1V; X = V, Y = R).

## Resumen

El grupo *guarani* del género *Drosophila* (Diptera: Drosophilidae) está formado por 24 especies cuyas relaciones no están claras. En el presente estudio se describen los cariotipos de *D. sachapuyu* Peñafiel and Rafael, 2018 and *D. zamorana* Peñafiel and Rafael, 2018, dos especies andinas miembros del grupo *guarani*. Se obtuvieron los cromosomas mitóticos a partir de los ganglios cerebrales de larvas de tercer estadío a través de la técnica de shock térmico y suspensión celular. El cariotipo de *D. sachapuyu* presenta 2n = 10 (4R, 1V; X = R, Y = R) mientras que *D. zamorana* presenta un cariotipo 2n = 12 (5R, 1V; X = V, Y = R).

The *guarani* group of *Drosophila* genus (Diptera: Drosophilidae) is a neotropical group proposed by Dobzhansky and Pavan (1943) and nowadays includes 24 species (Bachli 2021): *D. limbinervis* (Duda, 1925); *D. griseolineata* (Duda, 1927); *D. ornatifrons* (Duda, 1927); *D. maculifrons* (Duda, 1927); *D. nigrifemur* (Duda, 1927); *D. guaru* (Dobzhansky & Pavan, 1943); *D. subbadia* (Patterson, 1943); *D. guaraja* (King, 1947); *D. alexandrei* (Cordeiro, 1951); *D. araucana* (Brncic, 1957); *D. huilliche* (Brncic, 1957); *D. tucumana* (Vilela and Pereira, 1985); *D. urubamba* (Vilela & Pereira 1993); *D. ecuatoriana* (Vela & Rafael, 2004); *D. pichinchana* (Vela & Rafael, 2004); *D. quitensis* (Vela & Rafael, 2004); *D. cuscungu* (Vela & Rafael, 2005); *D. butantan* (Ratcov et al., 2017); *D. sachapuyu* (Peñafiel & Rafael, 2018); *D. zamorana* (Peñafiel & Rafael, 2018); *D. quinarensis* (Peñafiel & Rafael, 2018); *D. caxarumi* (Peñafiel & Rafael, 2018); *D. misi* (Peñafiel & Rafael, 2018); *D. peixoto* (Vaz et al., 2018).

Some misidentification have occurred in this group, *D. guarani* was synonymized to *D. ornatifrons*, *D. guaramunu* was synonymized to *D. maculifrons* (Vilela and Bächli 1990) and *D. pulla* (Pavan and Cunhas, 1947) was synonymized to *D. guaraja* (Freire-Maia and Pavan 1950). Also, *D. peruensis* was classified in the *guarani* group and posteriorly was moved to the *peruensis* group which includes spotted thorax species (Ratcov and Vilela 2007), and *D. amplipennis* (Malloch, 1934) was proposed like an aberrant member (Vilela and Bachli 2004), however it is not included in *guarani* group (Bachli 2021).

The *guarani* group was divided into two subgroups based on the shape of X chromosome (V or rod shape) (King 1947) but nowadays, the *guarani* subgroup has been accepted and the *guaramunu* subgroup has been dismissed (Vaz et al. 2018). Phylogenetic relationships of *guarani* group, based on morphology, cytology, and molecular markers suggested that species of *guarani* and *tripunctata* group are related and that *guarani* group is not monophyletic (Throckmorton 1962, Robe et al. 2002).

In this work are described the karyotypes of *Drosophila sachapuyu* and *Drosophila zamorana*, two Andean species that recently have been included in the *guarani* group based on the external morphology and terminalia (Peñafiel-Vinueza and Rafael 2018). Additionally, we compile the information about the karyotype of ten species of *guarani* group.

## Materials and Methods

### *Drosophila* Stocks

Were analyzed natural populations of *D. zamorana* (QCAZ-I 3266) (location 3°59′16.7ʺS; 79°5′35ʺW) and *D. sachapuyu* (QCAZ-I 3309) (4°6′53.7ʺS; 79°10′54.6ʺW) collected in the Podocarpus National Park (Zamora Chinchipe and Loja provinces in Ecuador) (Peñafiel and Rafael 2018) and maintained in the *Drosophila ceparium* of Evolutionary Genetics Laboratory of Pontificia Universidad Católica del Ecuador. The species are maintained in banana culture medium supplemented with fresh fruit, in temperate room at 17°C, 12 h cycle light/dark.

### Cytological Preparations

Third instar larvae (10 males, 10 females) of *D. sachapuyu* and *D. zamorana* stocks were dissected to obtain the cerebral ganglia and to observe metaphasic nucleus. The male larvae are identified by the presence of the testes (transparent balls) in the last segments of the larva. Chromosomal plates were prepared for cell suspension method (Cardoso and Dutra 1979) and thermic shock (Holmquist 1975), stained with Giemsa and posteriorly the C banding was performed.

Ten metaphasic nucleus for each sex and species were observed and the chromosome number was counted using the Ziess Axioskop 2 plus – HAL 100 microscope,100× objectives lenses, and optovar 2×. Pictures were obtained with a Cannon PowerShot A640 camera. The modal number was considered the chromosome number of the species.

Centromeric index (CI), centromeric ratio (CR), and relative length (RL) of the chromosomes were estimated using the Axio Vision 4.4. Standard deviation of relative length was analyzed using the SPSS statistical package 26.0v.

## Results

### Karyotype of *D. zamorana*

**Fig. 1.**
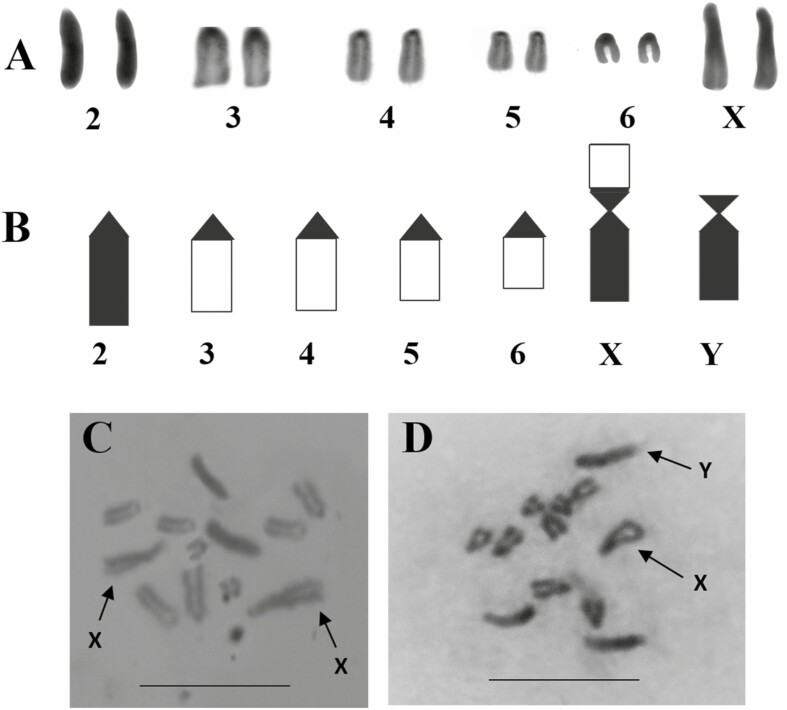
*D. zamorana* (2*n* = 12): A) karyotype, B) Idiogram, C) Female metaphase plate, D) Male metaphase plate. Scale bar = 5 µm.

The chromosome number of *D. zamorana* was 2*n* = 12 (5R, 1V) (X = V, Y = R), five autosomes, and the sexual pair (Fig. 1).

The pair 2 rod-shaped, telocentric, completely heteropycnotic. The pairs 3, 4, 5, and 6, all of them are rod-shaped, telocentric, and heterochromatic in the centromeric region. The chromosome X is V-shaped, submetacentric, heterochromatic in the proximal region of p arm and completely the q arm. The chromosome Y is rod-shaped, telocentric, and completely heterochromatic (Table 1).

**Table 1.** *D. zamorana* chromosome measurements and morphology

Chromosome	TL (µm)	σ (*n* = 10)	RL (%)	CI	Morphology
X	2.77	0,24	24.58	0.29	Submetacentric
2	1.8	0,21	15.93	0.07	Telocentric
3	1.63	0,19	14.43	0.07	Telocentric
4	1.47	0,22	13.03	0.07	Telocentric
5	1.22	0,2	10.83	0.08	Telocentric
6	0.97	0,14	8.57	0.1	Telocentric
Y	1.43	0,03	12.63	0.1	Telocentric

TL: Total Length, RL: Relative Length, CI: Centromeric Index, σ: St. Deviation.

### Karyotype of *D. sachapuyu*

**Fig. 2.**
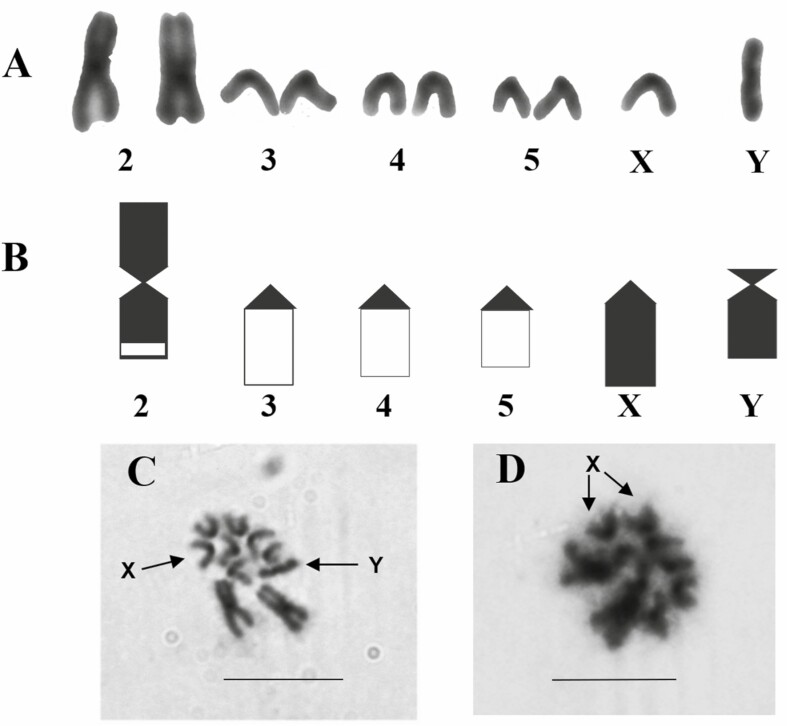
*D. sachapuyu* (2*n* = 10): A) karyotype, B) Idiogram, C) Female metaphase plate, D) Male metaphase plate. Scale bar = 5 µm.

The chromosome number of *D. sachapuyu* is 2*n* = 10 (4R, 1V) (X = R, Y = R), four autosomes, and sexual chromosomes (Fig. 2).

The pair 2, V-shaped, submetacentric, the proximal region of p arm, and almost the total length of q arm are heterochromatic. The pairs 3, 4, and 5, all of them are rod-shaped, telocentric, and heterochromatic in the pericentromeric region of large arms. The chromosome X is rod-shaped, telocentric, heterochromatic in the centromeric region, and p arm. The chromosome Y is rod-shaped, telocentric, and totally heterochromatic (Table 2).

**Table 2.** *D. sachapuyu* chromosome measurements and morphology

Chromosome	TL (µm)	σ (*n* = 10)	RL	CI	Morphology
2	3.37	0,45	29.18	0.3	Submetacentric
X	2.08	0,22	18.09	0.06	Telocentric
3	1.82	0,21	15.81	0.06	Telocentric
4	1.63	0,16	14.17	0.06	Telocentric
5	1.44	0,21	12.48	0.07	Telocentric
Y	1.1	0,05	10.27	0.08	Telocentric

TL: Total Length, RL: Relative Length, CI: Centromeric Index, σ: Standard deviation.

Karyotype description of *D. zamorana* and *D. sachapuyu* is added to the 10 karyotypes previously described for the species of *guarani* group (Table 3).

**Table 3.** Karyotype characteristics of 12 species of *guarani* group

Species	2*n*	Karyotype	Chromosomes		References
			X	Y	
*D. guaru (Dobzhansky and Pavan 1943)	12	4R, 1V, 1D	V	J	Dobzhansky and Pavan 1943, King 1947
*D. ornatifrons (Duda 1927)	12	5R, 1V	V	V	Dobzhansky and Pavan 1943, King 1947
*D. subbadia(Patterson 1943)	12	5R, 1V	V	J	King 1947
*D. maculifrons* (Duda 1927)	12	5R, 1D	R	R	Dobzhansky and Pavan 1943, King 1947
*D. griseolineta* (Duda 1927)	12	5R, 1D	R	R	Dobzhansky and Pavan 1943, King, 1947
*D. araucana* (Brncic 1957)	12	5R, 1D			Brncic 1957
*D. limbinervis* (Duda 1925)	12	5R, 1D	R	R	Clayton and Wasserman 1957
*D. zamorana* (Peñafiel and Rafael 2018)	12	5R, 1V	V	R	Authors
*D. guaraja* (King 1947)	10	3R, 1V, 1D / 3R, 1V	R	R	Pavan and da Cunha 1947, King 1947
*D. butantan* (Rarcov et al. 2017)	10	2R, 2V, 1D	V	R	Ratcov et al. 2017
*D. sachapuyu* (Peñafiel and Rafael 2018)	10	4R, 1V	R	R	Authors
*D. alexandrei* (Cordeiro 1951)	8	3R, 1V			Cordeiro 1951

*Species members of *guarani* subgroup; V: metacentric chromosome, J: submetacentric or subtelocentric chromosome, R: telocentric chromosome, D: dot chromosome.

## Discussion

Previous studies of *guarani* group based on morphology, chromosomes, and molecular phylogenies suggested that the relationships among the species included in this group are not clear. In the *guarani* group are included species with variation in morphology, brownish and spotted thorax (Vilela and Pereira 1985), heterogeneity in terminalia structures (Throckmorton 1962), and different chromosome number (Kastritsis et al. 1970). These characteristics are shared with some species of *tripunctata* group, therefore these variations open some questions respect the relationships among the species of *guarani* and *tripunctata* groups.

The phylogeny proposed by Throckmorton’s (1962) based on the external morphology and the anatomical characteristics of genital structures place the *tripunctata* and *guarani* group very close in the tree. This near relationship between *guarani* and *tripunctata* species is supported posteriorly by Kastritsis (1969) and Kastritis et al. (1970), they proposed the division of *guarani* group in two species groups based on the similarity of banding patterns of polytene chromosomes between *D. griseolineata* (*guaramunu* subgroup) and *D. mediostriata* (Duda 1925) (karyotype 2*n* = 12; 5R, 1D) (*tripunctata* group) (Dobzhansky and Pavan 1943).

Also, King (1947) proposed the division of *guarani* group in two subgroups based on the shape of Y chromosome: the *guarani* subgroup with V shape (*D. guaru*, *D. ornatifrons*, and *D. subbadia*) and *guaramunu* subgroup with rod shape (*D. maculifrons*, *D. griseolineta*, and *D. guaraja*). Recently Vaz et al. (2018) based on chromosome analysis dismissed the *guaramunu* subgroup, therefore only the *guarani* subgroup is accepted in the *guarani* group.

With the description of the karyotypes of *D. sachapuyu* and *D. zamorana*, there is available information about the karyotype of 12 species of *guarani* group (Dobzhansky and Pavan 1943, King 1947, Pavan and Cunha 1947, Clayton and Wasserman 1957, Vilela and Bächli 1990, Ratcov et al. 2017, Vilela and Pereira 1985, Cordeiro 1951, Brncic 1957) which shown variation in chromosome number from 2*n* = 8 to 2*n* = 12 and four types shapes and morphologies: V-shaped (V), J-shaped (J), rods (R), and dots (D) (King 1947, Ratcov et al. 2017, Cordeiro 1951), which correspond V for a metacentric chromosome, J for a submetacentric or subtelocentric chromosome, R for a telocentric chromosome, and D for dot, a small or very small chromosome (Deng et al. 2007). Species of *guarani* group present the Y chromosome in rod shape, except *D. guaru*, *D. subbadia*, and *D. ornatifrons*. The X chromosome could be V or rod shape (King 1947) (Table 3).

According to Vaz et al. (2018) in the *guarani* subgroup has been included four species (*D. ornatifrons*, 5R, 1V; *D. subbadia*, 5R, 1V; *D. guaru* 4R, 1V, 1D; and *D. peixoto*). Three of them present the same chromosome number 2*n* = 12, however each species showed different shape of sexual chromosomes. *D. ornatifrons* and *D. subbadia* present karyotype 5R, 1R, but the morphology of Y chromosome is V-shaped in *D. ornatifrons* and J-shaped in *D. subbadia*. Similar morphology (V-shaped) of sexual chromosomes X have the species *D. guaru* and *D. ornatifrons*, however *D. guaru* presents the Y chromosome J-shaped and a dot chromosome that is not present in *D. ornatifrons* neither in *D. subbadia*. An important characteristic described only for *D. ornatifrons* is the presence of satellites in all the rod chromosomes. There is no information available about the karyotype of *D. peixoto*, the fourth member of *guarani* subgroup.

The rest of species remain in the *guarani* group but have not been assigned to a subgroup. Five species present a karyotype 2*n* = 12, three of them, *D. maculifrons* (5R, 1D), *D. griseolineata* (5R, 1D), *D. araucana* (5R, 1D), present a dot chromosome, while *D. zamorana* (5R, 1V) not present the dot chromosome similar to *D. limbinervis* (5R, 1D). On the other hand, *D. griseolineata* presents X and Y chromosome large rod-shaped, however the Y chromosome presents a satellite. While, *D. maculifrons* and *D. zamorana* (5R, 1V) present V-shaped and rod-shaped X chromosome, respectively, and rod-shaped Y chromosome.

*D. guaraja* (3R, 1V, 1D), *D. butantan* (2R, 2V, 1D), and *D. sachapuyu* (4R, 1V) present karyotype 2*n* = 10, all of them present a large V-shaped autosome but only *D. guaraja* and *D. butantan* present a dot chromosome. Respect the sexual chromosomes, *D. guaraja* and *D. sachapuyu* show rod-shaped X and Y chromosomes, however the X chromosome of *D. sachapuyu* is two times longer the Y chromosome, while the X chromosome of *D. guaraja* is a short rod. In *D. butantan*, the X chromosome is V-shaped and the Y chromosome is rod-shaped.

Only the species *D. alexandrei* (3R, 1V) presents a karyotype 2*n* = 8, which includes a large V-shaped autosome (similar to the species 2*n* = 10), and dot chromosome is absent. The X and Y chromosomes are rod-shaped, similar size to autosomes.

Molecular phylogeny of *guarani* group supports the monophyly only for the *guarani* subgroup (*D. subbadia* and *D. ornatifrons*) while the *guarani* group is not a monophyletic group (Robe et al. 2002, 2005, Izumitani et al. 2016). In two molecular studies has been proposed a *tripunctata* lineage cluster which includes species of *guarani* (*D. ornatifrons*, *D. griseolineata*, and *D. maculifrons*), *tripunctata*, and *cardini* groups (Robe et al. 2002, Yotoko et al. 2003). Also, a molecular phylogeny of *tripunctata* radiation, based on mitocondrial genes, suggested the existence of four clades, one of them is the clade *tripunctata-guaramunu* lineage conformed by the species *D. griseolineata*, *D. maculifrons* (*guarani* group), *D. frotapessoai* (Vilela and Bachli 1990), and *D. paramediostriata* (Townsend and Wheeler 1955) (karyotype 2*n* = 12; 5R, 1D) (*tripunctata* group) (Hatadani et al. 2009). In other molecular analysis, few species of *guarani* group has been included however in all of them is observed that species of *guarani* and *tripunctata* groups are closely related (Izumitani et al. 2016, Robe et al. 2005).

Comparison of karyotypes of *guarani* species showed differences in the size of autosomes but especially in the morphology and size of sexual chromosomes which in some species are twice the size of the autosomes (*D. maculifrons*, *D. griseolineata*, and *D. araucana*). This characteristic is described also for the species *D. paramediostriata* (Townsend and Wheeler 1955) of the *tripunctata* group. These four species present karyotype 2*n* = 12 and the molecular phylogenies proposed that are closely related, however are lacking information about more species to confirm the relationship of *guarani* group.

Morphological and phylogenetic analysis have not provided enough information to clarify the relationship of the *guarani* group. However, karyotype information and new genomic analysis could explain changes in chromosome number and chromosome structure to clarify the relationships of *guarani* group and the high radiation of *Drosophila* species in the Neotropical region.
